# Comparative transcriptome analysis of a taxol-producing endophytic fungus, *Aspergillus aculeatinus* Tax-6, and its mutant strain

**DOI:** 10.1038/s41598-020-67614-1

**Published:** 2020-06-29

**Authors:** Weichuan Qiao, Tianhao Tang, Fei Ling

**Affiliations:** Department of Environmental Engineering, College of Biology and the Environment, Nanjing Forestry University, Nanjing, 210037 Jiangsu Province China

**Keywords:** Metabolic engineering, Applied microbiology

## Abstract

Taxol is a rare but extremely effective antitumor agent extracted from *Taxus yew* barks. *Taxus* plants are valuable and rare species, and the production of taxol from them is a complex process. Therefore, taxol-producing endophytic fungi seem to be a promising alternative because of their high practical value and convenient progress. In this study, the transcriptome of an endophytic fungus, *Aspergillus aculeatinus* Tax-6 was analyzed in order to understand the molecular mechanisms of producing fungal taxol. The results showed that genes involved in the mevalonate (MVA) pathway and non-mevalonate (MEP) pathway were expressed, including isopentenyl pyrophosphate transferase, geranyl pyrophosphate transferase, and geranylgeranyl pyrophosphate synthetase. However, those downstream genes involved in the conversion of taxa-4(5)-11(12)-diene from geranylgeranyl pyrophosphate were not expressed except for taxane 10-beta-hydroxylase. Additionally, a mutant strain, *A. aculeatinus* BT-2 was obtained from the original strain, *A. aculeatinus* Tax-6, using fungicidin as the mutagenic agent. The taxol yield of BT-2 was 560 µg L^−1^, which was higher than that of Tax-6. To identify the mechanism of the difference in taxol production, we compared the transcriptomes of the two fungi and explored the changes in the gene expression between them. When compared with the original strain, Tax-6, most genes related to the MVA pathway in the mutant strain BT-2 showed upregulation, including GGPPS. Moreover, most of the downstream genes were not expressed in the mutant fungi as well. Overall, the results revealed the pathway and mechanism of taxol synthesis in endophytic fungi and the potential for the construction of taxol-producing genetic engineering strains.

## Introduction

Taxol, one of the taxanes, can be used for curing tumors as an integration in multidrug regiments^1,2^. Formerly, taxol could be obtained from *Taxus chinensis*, but since the trees are a rare plant species, the concentration of taxol in the extract is only 0.01–0.03%^3^. Thus, chemical synthesis has been explored for taxol production^4,5^. However, the technologies are inhibited for practical use owing to their complicated and expensive processes, which has led researchers to focus on biological methods^6–8^. Endophytic fungi in yew trees can be used for biological fermentation of taxol production, and has been observed to be a promising method because the fungi are widely available and taxol is produced conveniently by fermentation^9^.

To date, numerous endophytic fungi have been found to produce taxol, the hosts of which are the *Taxus* spp., *Platycladus orientalis* (L.), *Wollemia pine*, *Torreya grandifolia*, *Terminalia arjune*^10–13^. However, the taxol concentration of most of the microbial fermentation solutions is lower than 1 mg L^−1^, which makes it difficult to be used in industrial applications^9^. In view of this shortcoming, several methods have been proved to increase the produced amount of fermentation taxol^14^. For example, a mutant strain was obtained from taxol-producing fungi, which showed a production of 225.2 µg L^−1^ vs. 20 µg L^−1^ in the original strain^15^. Additionally, some chemicals can induce an *Aspergillus niger* strain to improve the taxol production by two folds^16^. However, the production concentration of taxol is still unsatisfactory; therefore, studies have been conducted to use molecular biotechnology to modify the expression of genes involved in taxol biosynthesis. Apart from these, genome shuffling has been conducted to increase taxol production^17^. Ajikumar et al. developed a novel method to promote taxol production via multivariate-modular engineering in the metabolic-pathway. The titers of taxadiene, the first committed taxol intermediate, increased to 1 g L^−1^ in *Escherichia coli*^18^. Nevertheless, only very few successful studies have been reported, primarily because of the lack of molecular information regarding the biosynthesis of taxol in fungi^9^.

Next generation sequencing is an advanced technology for complex transcriptome analysis, which allows for a quick and comprehensive insight into gene structure, facilitating the recognition of target genes and the analysis of gene expression of an organism^19,20^. De novo assembly performs well for short read sequence data and has been widely used to investigate the synthesis of taxol in *Taxus*. Qiao et al.^21^ analyzed the transcriptome of *Cephalotaxus hainanensis* and found a large number of gene numbers involved in taxol synthesis. Sun et al.^22^ conducted comparative transcriptome analysis between non-elicited *Taxus* X media and methyl jasmonate-elicited cultures and found that some genes were involved in the synthesis of terpenoid backbone and the bioconversion steps from geranyl pyrophosphate to 10‐deacetyl Baccatin III. However, to the best of our knowledge, investigations of the taxol biosynthesis pathway and taxol-related genes in taxol-producing endophytic fungi remain unclear to date^23^.

In our previous study^24^, we isolated a taxol-producing endophytic fungus, *Aspergillus aculeatinus* Tax-6, from *Taxus* X Media*,* and researched a method for enhancing taxol production. In this study, we obtained a mutant strain with a higher taxol production from Tax-6 to comprehensively understand the taxol synthesis pathway in endophytic fungi. By comparing the gene expression of the wild-type and mutant strains of Tax-6, more than 60 genes involved in taxol synthesis and transcriptome factors that may influence the taxol biosynthesis pathway were obtained. We also found that the mutant strain performed better under environmental stress.

## Materials and methods

### Taxol-producing strain

The taxol-producing endophytic fungus *A. aculeatinus* Tax-6 (CCTCC M 2,016,614) was isolated from the bark of *Taxus* X Media^24^.

### Extraction of fungal taxol

The extraction of fungal taxol followed the method reported by Wang, et al.^16^. First, the endophytic fungus was aerobically cultured in potato dextrose broth medium at 28 °C for eight days. The entire culture was passed through four layers of cheesecloth, after which the culture fluid was collected and extracted three times (about two hours each time), then further purified with 1/10 volume of hexane. All of the organic phase were collected and then dried at 45 °C by rotary evaporator (Buchi R-200, Switzerland). Finally, the residue was dissolved in 1 mL of methanol and filtered through a 0.2 μm polymeric filter.

### Determination of fungal taxol

A C18 column (250 mm × 4.4 mm × 10 μm) was used for separation of taxol production on a Dionex Ultimate-3000 HPLC system. The mobile phase consisted of methanol: water (70: 30) applied at a flow rate of 1 mL min^−1^ and a column temperature of 40 °C. The registration of the peak and retention time was recorded by reading the UV absorbance at 227 nm. The fungal taxol was quantified by comparing the peak area to that of a standard sample (standard taxol was diluted to 0.2, 0.4, 0.6, 0.8 and 10 mg L^−1^ by methanol). The HPLC chromatogram including the sample and the standard compound of taxol have been provided in our previous study^24^.

### Mutagenesis assay of taxol-producing strain

A chemical mutagenesis assay was applied to obtain mutant fungus with more taxol production using mycostatin as the mutagen. *A. aculeatinus* Tax-6 was cultured in potato dextrose agar (PDA) medium with a certain concentration of mycostatin (CAS:1400-61-9, Sigma-Aldrich ) solution on a culture dish. After five days, the colonies were picked out from the dishes in which the fungi had high fatality rates (90–95%) were picked out from the dishes, and then introduced by the streak plate method into a fresh PDA medium to culture for five days again. The selected fungus was repeatedly purified (five times) through inoculation before the mutant strain was obtained. The mutant fungus with the highest taxol yield was then named BT-2.

### Transcriptome analysis

#### RNA extraction

Total RNA of the two fungi was extracted by the CTAB method according to the protocol of the RNA extraction kit (Invitrogen 15596-026). The residue of DNA was removed by DNase (Sangon Biotech, China). Qubit2.0 (Q32866, Invitrogen, USA) was used to measure the concentration of the extracted RNA, and then gel electrophoresis (DYY-11, LiuYi Instrument, China) was used to test the genome contamination and integrity of the extracted RNA. Each experiment had three biological replicates for the transcriptome sequencing (RNA-seq).

#### Library construction and Illumina sequencing

The preparation of the transcriptome library of the two fungi was conducted by Sango Biotechnology (Shanghai, China). A VAHTS™ mRNA-seq V2 Library Prep Kit for Illumina® (Vazyme Biotech Co., Ltd, China) was used to construct an RNA-seq library according to the manufacturer’s protocol. Briefly, an amount of 200 ng of the total RNA sample was bound to oligo-dT attached magnetic beads to select poly-A mRNA. The selected mRNAs were fragmented to 150–500 bp at high temperature in the presence of magnesium ions. The cleaved mRNA fragments were then used to synthesize first strand cDNA using reverse transcriptase and random hexamer primers. Second strand cDNA synthesis followed, using DNA Polymerase I and RNase H. The cDNA products were purified with VAHTS DNA Clean Beads, end-repaired and A-tailed using End Rep Mix and dA-Tailing Buffer Mix, and then ligated with RNA Adapters. The library was amplified by PCR, and its quality was checked on Agilent 2,100 Bioanalyzer using Agilent DNA 1,000 kit (Agilent, Cat. No. 5067-1504). The bioanalyzer results should reveal a peak of products at the size expected based on the average fragment size and length of the adaptors. Each single cDNA library was sequenced using Illumina HiSeq 2000 (Illumina, San Diego, CA, USA) and a v2.5 TruSeq Paired End HiSeq flow cell/cluster kit (Illumina, San Diego, CA, USA) according to the manufacturer’s instructions^25^. The length of paired end was 2 × 150 bp.

#### De novo assembly

Prinseq v 0.19.5 (https://prinseq.sourceforge.net/) was used to control the quality of the raw reads. The sequencing connectors were removed first by Cutadapt (https://pypi.python.org/pypi/cutadapt/1.2.1), and the bases were removed when the quality of their read tails was lower than 20, and then the N-containing sequences (< 35 bp) were cut before the assessment of sequences contamination. De novo assembly was employed to construct the transcripts because of the absence of a reference genomic sequence. The raw reads of the samples after quality control were combined, and then were assembled de novo using Trinity v r20140717) (https://trinityrnaseq.sourceforge.net/)^26^. The overlapping reads with specific length (> 200 bp) were first assembled into contigs, after which the reads were mapped back onto the contigs using RSeQC v 2.6.1) (https://rseqc.sourceforge.net/) with the paired-end method and gap-filling in silico. Chimeric reads were subsequently excluded from the missing assembly using three or more read pairs as the criterion to define order and distance between two contigs. This step was able to detect the contigs from the same transcript and could be used to calculate the distance between two contigs. The contigs were then assembled to obtain longer sequences, but this step ended when constructed sequences could not be extended any more by the Trinity software and they were considered unique transcripts. Finally, the unique genes were obtained using the sequence-splicing redundancy removal routine to process the unique transcripts.

#### EST-SSR detection and primer design

The unique genes and transcripts were screened for SSR markers using MISA (https://pgrc.ipk-gatersleben.de/misa/). The parameters were adjusted for identification of perfect mononucleotide, di-nucleotide, tri-nucleotide, tetra-nucleotide, pentanucleotide, and hexa-nucleotide motifs with a minimum of 10, 6, 5, 5, 5, and 5 repeats, respectively^27,28^. The primers were designed by primer3 using default parameters (https://primer3.sourceforge.net/).

#### Transcriptome sequencing data analysis

The unigene gene sequences were blasted using NCBI blast 2.2.28 + software with the NR (NCBI Non-redundant Protein Sequences), NT (NCBI Nucleotide Sequences), KOG (EuKaryotic Ortholog Groups), CDD (Conserved Domain Database), PFAM (Protein Family), Swissprot (a manually annotated and reviewed protein sequence database), TrEMBL, GO (Gene Ontology), and KEGG (Kyoto Encyclopedia of Genes and Genomes) libraries^29–31^, respectively. All the annotation details with a similarity > 30% and E < 1 × e^−5^ were obtained through the combination of the unigene. To classify the functions of genes obtained, BLASTX searches of the NR database, the SwissProt database and TrEMBL were carried out using an E-value cutoff of 1 × e^−5^^32^.

Based on the annotation in the NR database, the Blast2Go program (https://www.blast2go.com/b2ghome) was employed to classify the gene functions into molecular functions, biological processes and cellular components^32^. After retrieving the associated GO terms, the possible functions of unigenes were predicted against KOG database. In parallel, the KEGG database (https://www.genome.jp/kegg) was used to conduct the pathway assignments of the unique genes using BLASTX^29^.

To investigate the response of chemical mutation on original fungus Tax-6, Fragments Per Kilobase of transcript per Million fragments mapped (FPKM) was used as an index to measure the gene expression level using the RSEM software, which can quantify transcript abundance from RNA-seq data without a reference genome^33^, after which we conducted false discovery rate (FDR) calculation and statistical analysis by EdgeR^34^. We defined differential expressed gene (DEG) as a gene with twofold expression changes between *A. aculeatinus* Tax-6 and BT-2 samples and FDR of less than 0.05.

### qRT–PCR analysis

The qRT–PCR experiments were used for detecting the expression levels of six genes involved in taxol biosynthesis^35^, which were performed using gene-specific primers with a QuantiFast SYBR Green PCR Kit (Qiagen) on a Bio-Rad T100™ Thermal Cyeler Real-Time PCR Detection System (Bio-Rad)^36^. The primer sequences were designed on two consecutive exons and the amplification efficiencies are shown in Table S1. The real-time melting curve and agarose gel electrophoresis were used to check the specificity of the primers^36^. qRT-PCR reactions were performed in 20 μL volumes containing 2 μL of diluted cDNA, 0.4 μL of 10 μM forward primer, 0.4 μL of 10 μM reverse primer, and 10 μL of 2 × SybrGreen qPCR Master Mix (Applied Biosystems). The thermal conditions of the qRT-PCR reactions were 95 °C for 3 min, and 45 cycles of 95 °C for 15 s, 57 °C for 20 s, and 72 °C for 30 s^37^. The relative expression levels were calculated using the 2^−∆∆Ct^ method^38^. GAPDH was used as the housekeeping gene. Each experiment was performed with three biological and technical replicates^24^. Analysis of variance (ANOVA) was used to examine differences in mean values. The 95% confidence interval (*p* < 0.05) was set as the significance threshold, which was used to evaluate the significance of the difference in gene expression between strains Tax-6 and BT-2^36^.

## Results

### Growth and taxol yield of the mutant strain BT-2

**Figure 1:**
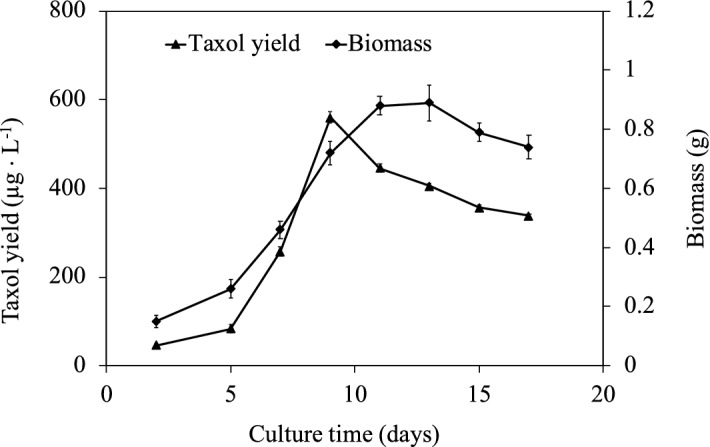
Taxol yield and biomass of the mutant strain BT-2 cultured in PBD medium for different culture time.

In this study, mycostatin was used to induce the genetic mutation of the strain Tax-6. As shown in Fig. S1, mycostatin can inhibit the growth of the original strain Tax-6, and the strain can’t grow when mycostatin concentration in the medium was 15 mg L^−1^. The mutant strain BT-2 was selected after the genetic mutation of mycostatin to Tax-6. The highest taxol yield of BT-2 was 560 μg L^−1^ on day 8, which was higher than that of Tax-6^24^ (Fig. 1).

### Sequencing and assembly

The transcriptomes of original strain *A. aculeatinus* Tax-6 and its mutant *A. aculeatinus* BT-2 were sequenced with Illumina sequencing technology to generate 55,976,870 and 68,241,536 raw reads, respectively (Table 1). The coverage of sequencing is shown in Fig. S2. The coverage was calculated by samtools (https://samtools.sourceforge.net/), which could reflect the proportion of fully detected and undetected genes in each sample, and could find whether there were more specific genes among samples. After strict quality control, 55,898,665 and 68,048,645 clean reads were obtained from *A. aculeatinus* Tax-6 and BT-2, and were combined before being assembled to 45,242 unigenes. The N50 and mean size of the combined unigenes were 2029 bp and 995 bp, respectively (Table S2). The length distribution of unigenes from 200 to more than 2000 bp is shown in Fig. S3. The distribution of GC content of the combined unigenes and transcripts is shown in Fig. S4. The combined transcripts data from *A. aculeatinus* Tax-6 and BT-2 are also shown in Table S2. 50 species were specified to assess the pollution of the sequences. The results showed that the first few species were very close to the source *A. aculeatinus* (Table S3), which indicated that the sample had no obvious pollution although a small read number of other species were identified.

**Table 1:** The result of removing miscellaneous from the original data.

Strains	Before QC			After QC	
	Total reads	Base numbers (bp)	≥ Q20 (%)	Total reads	Base numbers (bp)
Tax-6	55,976,870	8,396,530,500	99.86	55,898,665	7,985,573,998
BT-2	68,241,536	10,236,230,400	99.72	68,048,645	9,871,570,321

### Functional annotation

**Figure 2:**
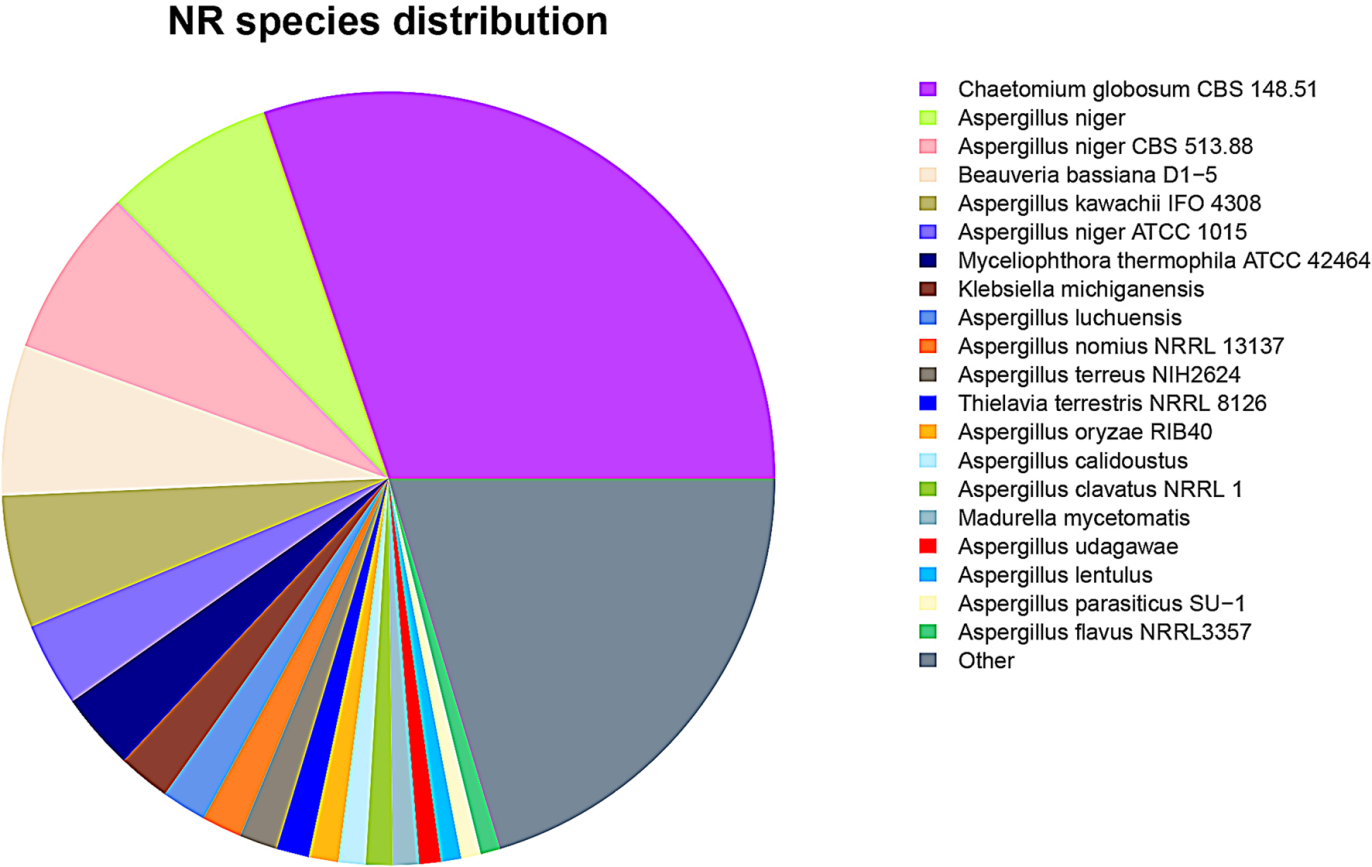
Percentage of top blast hits by species.

The proportion of annotation of each database is shown in Table S4. These annotations gave us a valuable resource for suggesting specific pathways and function genes in *A. aculeatinus* Tax-6. To classify the functions of genes obtained, BLASTX searches of the NR database were conducted using an E-value cutoff of 1.0 × e^−5^, accounting for 68.89% of all unigenes. Species distribution analysis revealed that *A. aculeatinus* Tax-6 had a number of homologous sequences with several species, and the genes from *Chaetomium globosum* had the highest similarity (30.23%), followed by *A. niger* (7.13%), *A. niger* CBS 513.88(7.08%), *Beauveria bassiana D1-5* (6.28%), and *A. kawachii IFO 4,308* (5.55%) (Fig. 2). In addition, 23,462 unique sequences (51.86%) were annotated into GO terms using Blast2Go, 5,555 (12.28%) of which were annotated with KEGG pathways using the Single-directional Best Hit (SBH) method^39^. Additionally, 22,474 unigenes (49.68%), 22,794 unigenes (50.38%), 19,462 unigenes (43.02%), 20,874 unigenes (46.14%), 14,410 unigenes (31.85%), 31,026 unigenes (68.58%) and 3,350 unigenes (20.17%) were annotated in the databases of CDD, NT, PFAM, Swiss-Prot, KOG and TrEMBL, respectively (Table S4). In total, 34,742 unigenes (76.79%) could be annotated with at least one database, and 3,765 unigenes (8.32%) could be annotated in all databases, with relatively good defined functional annotations.

**Figure 3:**
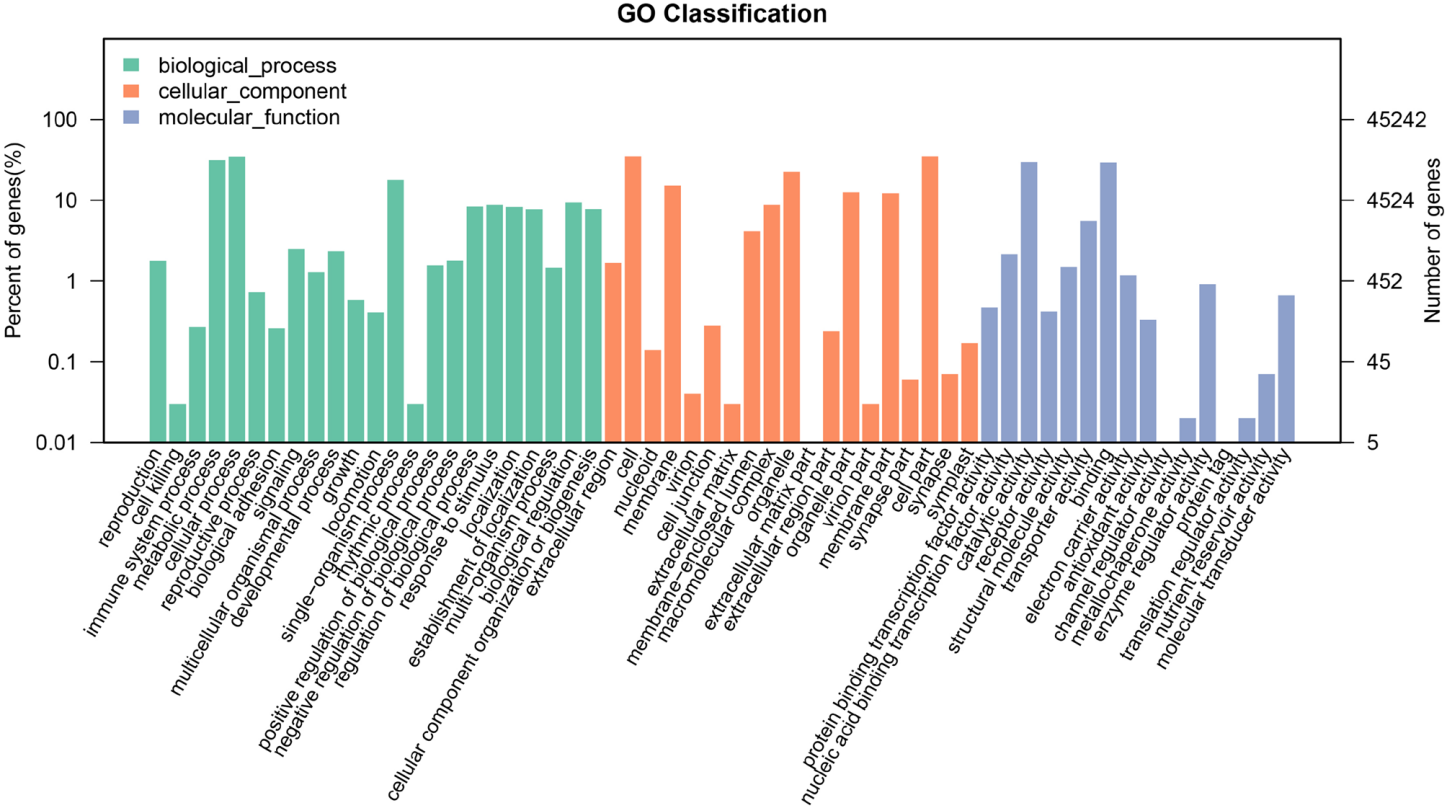
GO classification of Tax-6 and BT-2.

The GO classification data was combined from *A. aculeatinus* Tax-6 and BT-2. The obtained putative gene functions assigned by homology searches were classified into biological processes, cellular components and molecular function based on the results of Blast Uniprot (Fig. 3). The results showed that encoded proteins involved in biological processes ranked first, including metabolic processes, cellular processes and single-organism processes. For the cellular component domain, most of the unique sequences were located in the cell and cell parts. For the molecular function domain, most unigenes took part in catalytic activity and binding. The high number of genes involved in metabolic processes and cell parts indicated that the endophytic fungi could actively produce secondary metabolites like taxol.

**Figure 4:**
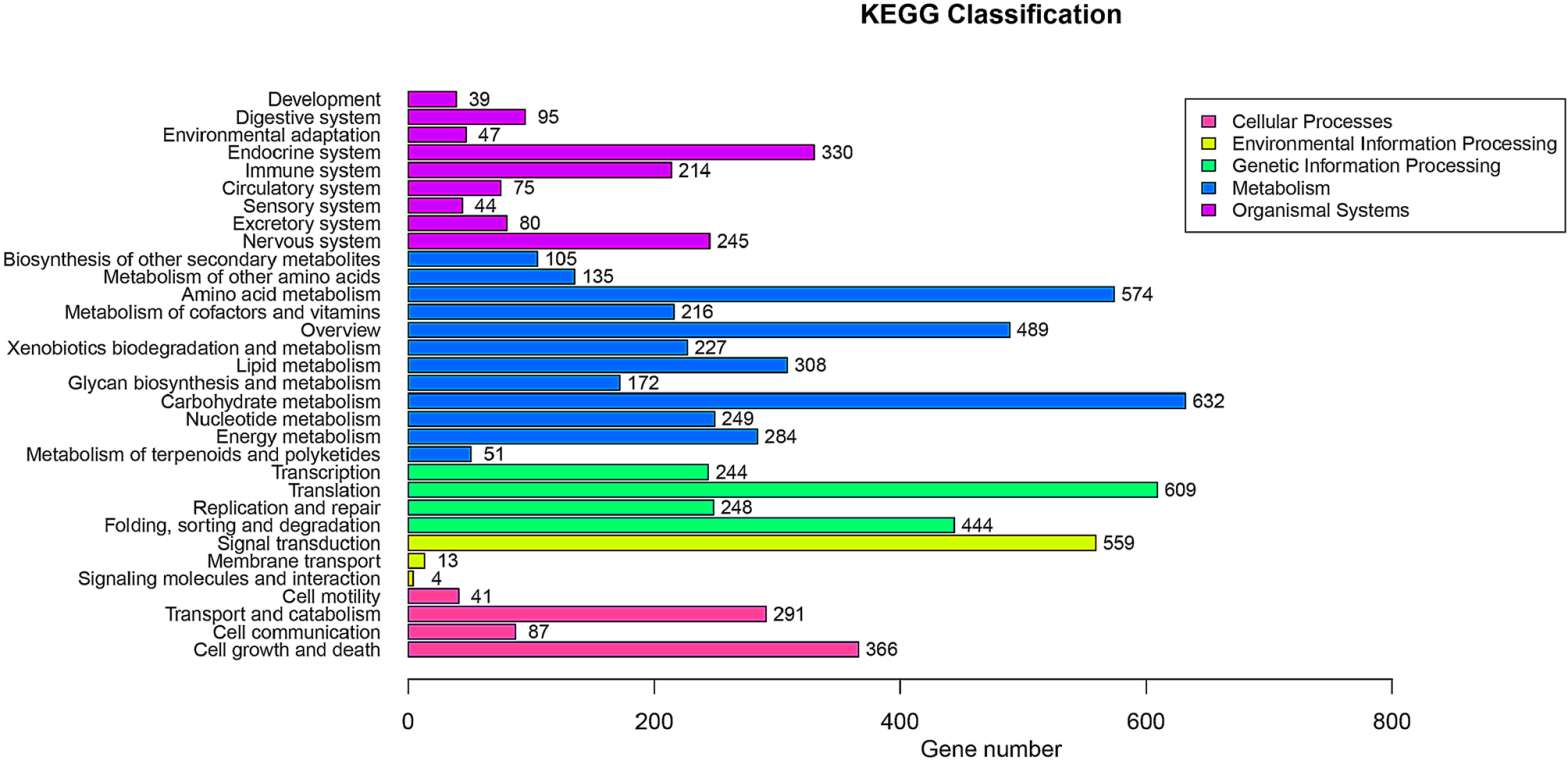
KEGG pathway classification^29–31^.

To understand the metabolic pathways in Tax-6 and BT-2, a BLASTX search against the KEGG database was used to map unique sequences to specific pathways as shown in Fig. 4. A total of 5,555 unique genes were annotated with the KEGG database, and the pathways were classified into cellular processes, environmental information processes, genetic information processes, metabolism and organismal systems. The largest unique pathways were carbohydrate metabolism, translation, signal transduction and amino acid metabolism.

### Genes involved in taxol synthesis in *A. aculeatinus* Tax-6

The biosynthesis pathway of taxol in *Taxus* plants has been revealed, which includes the synthesis of the precursor of terpenoid––isopentenyl pyrophosphate (IPP), carbon ring backbone––Baccatin III and side chains of taxol. However, the biosynthesis pathway of taxol originated from endophytic fungi was not clear. According to the KEGG annotation in this study, over 20 unique sequences were involved in terpenoid backbone biosynthesis (Table S5), including 1-deoxyxylulose 5-phosphate reductoisomerase (DXR), 4-hydroxy-3-methylbut-2-enyl diphosphate reductase (HDR), 3-hydroxy-3-methylglutaryl-coenzyme A synthase (HMGS) and 4-hydroxy-3-methylbut-2-enyl diphosphate reductase (HMDR). These included genes were related to IPP and dimethylallyl diphosphate (DMAPP) biosynthesis in the mevalonate (MVA) pathway and the non-mevalonate (2-C-Methyl-d-erythritol-4-phosphate, MEP) pathway^40,41^. In addition, genes responsible for geranyl pyrophosphate (GPP) and geranylgeranyl pyrophosphate (GGPP) synthesis were also found^42^, which are the important taxol precursor and can promote the production of taxol. However, genes involved in the conversion of taxa-4(5)-11(12)-diene from GGPP, the key step in taxol production, were not found (Table 1). Taxa-4(5)-11(12)-diene is a key carbon ring backbone of the taxol structure. The unique sequence was found to be homologous to taxane 10-β-hydroxylase (T10βH) involved in taxol synthesis by *Ozonium sp.* BT2, which is used in taxol biosynthesis and is known as P450 hydroxylase^43^.

### Comparison of the expression levels of genes between *A. aculeatinus* Tax-6 and BT-2

**Figure 5:**
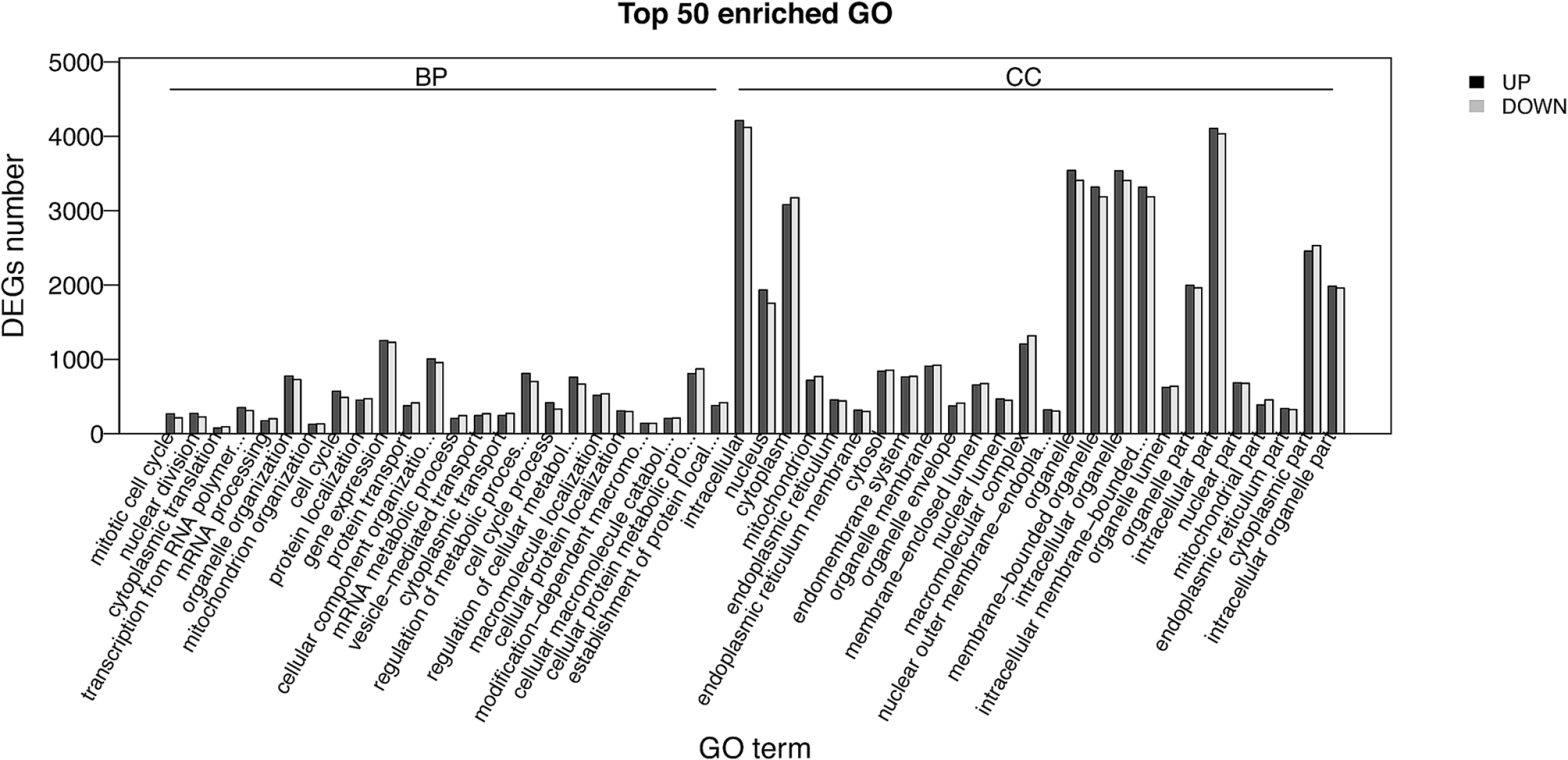
The different expression gene set (DEGs) number that were significantly different (*p* < 0.05) when comparing *A. aculeatinus* Tax-6 with BT-2.

As shown in Fig. 5, most of the genes involved in metabolic pathways in the mutant fungus, *A. aculeatinus* BT-2, changed when compared with that in Tax-6; moreover, there were 11,279 up-regulated genes and 8,097 down-regulated genes in BT-2 (Fig. S5). About 15.69% (3,041) of the unique genes showed changes of more than two folds in 312 pathways. The increase in pathways included terpenoid backbone biosynthesis, the cell cycle and the citrate cycle. Pathways that showed decreased gene abundance were related to phenylalanine metabolism, metabolism of xenobiotics by cytochrome P450 and base excision repair. As shown in Fig. S6, in the terpenoid backbone biosynthesis pathway, the MVA pathway and MEP pathway were both found^29–31^. When compared with the original fungus Tax-6, most genes showed up-regulated expression in this pathway in BT-2, including GPPS, which transferred the product of the dimethylallyl-PP pathway to GPP, the backbone of taxol. As shown in Fig. S7, when compared with Tax-6, the gene expression of phenylalanine biosynthesis changed in the phenylalanine metabolism pathway of BT-2^29–31^. According to Fleming et al.^44^, phenylalanine is converted to β-phenylalanine and then further to phenylisoserine, after which phenylisoserine is combined with the backbone of taxol (like Baccatin III) and forms the side chain of taxol. Fig. S8 shows that the expression of genes related to glycine, serine and threonine metabolism changed in BT-2 compared with Tax-6^29–31^. Most of these genes were up-regulated, which showed that doses of glycine, serine and threonine could promote taxol production. The result is consistent with the previous researches^24,45^.

**Figure 6:**
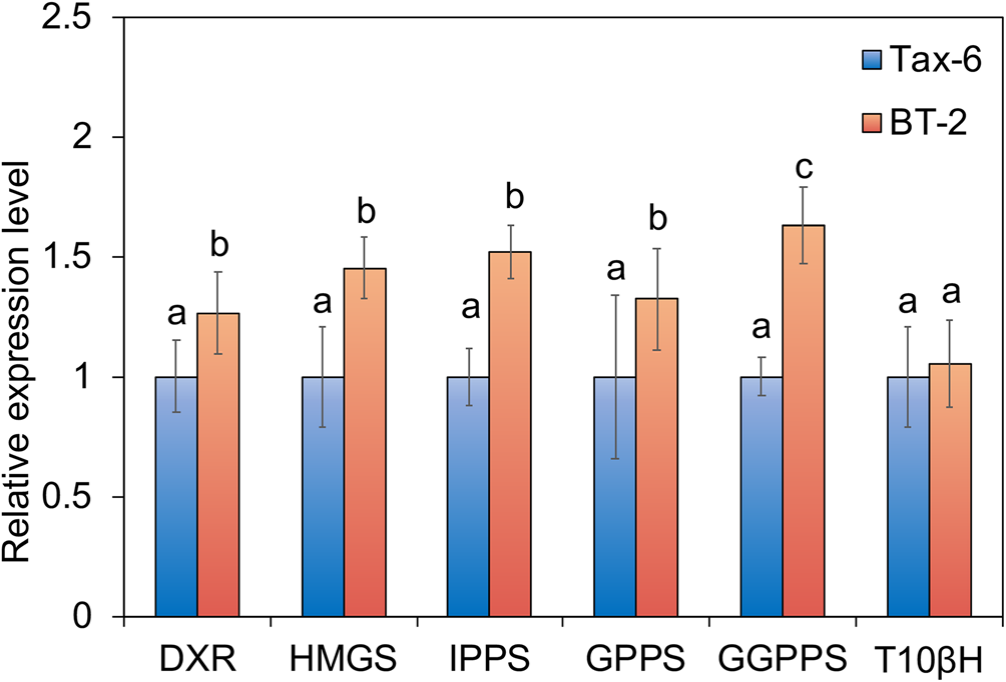
Comparison of transcriptome expression levels of genes involved in key enzymes involved in the biosynthetic pathway of paclitaxel between *A. aculeatinus* Tax-6 and BT-2. Values with different letters (**a**–**c**) differ significantly (*p* < 0.05). Error bars represent standard deviations (n = 3).

DXR, 1-hydroxy-3-methylglutaryl enzyme A reductase (HMGR), IPPS, GPPS, and geranylgeranyl diphosphate synthase (GGPPS) are the key enzymes involved in the MVA pathway and MEP pathway, and T10βH is used to catalyze taxadiene to Baccatin III. To find out effects of important enzymes on taxol biosynthesis, we compare the expression levels of DXR, HMGR, IPPS, GPPS,GGPPS and T10βH between Tax-6 and BT-2 by qRT-PCR. As shown in Fig. 6, compared to Tax-6, the expressions of genes involved in the six enzymes in BT-2 were all upregulated. However, compared with Tax-6, the expression of GGPPS in BT-2 was up-regulated more significantly than that of other enzymes (*p* < 0.01), whereas the change in T10βH expression was not obvious (*p* > 0.05).

## Discussion

**Figure 7:**
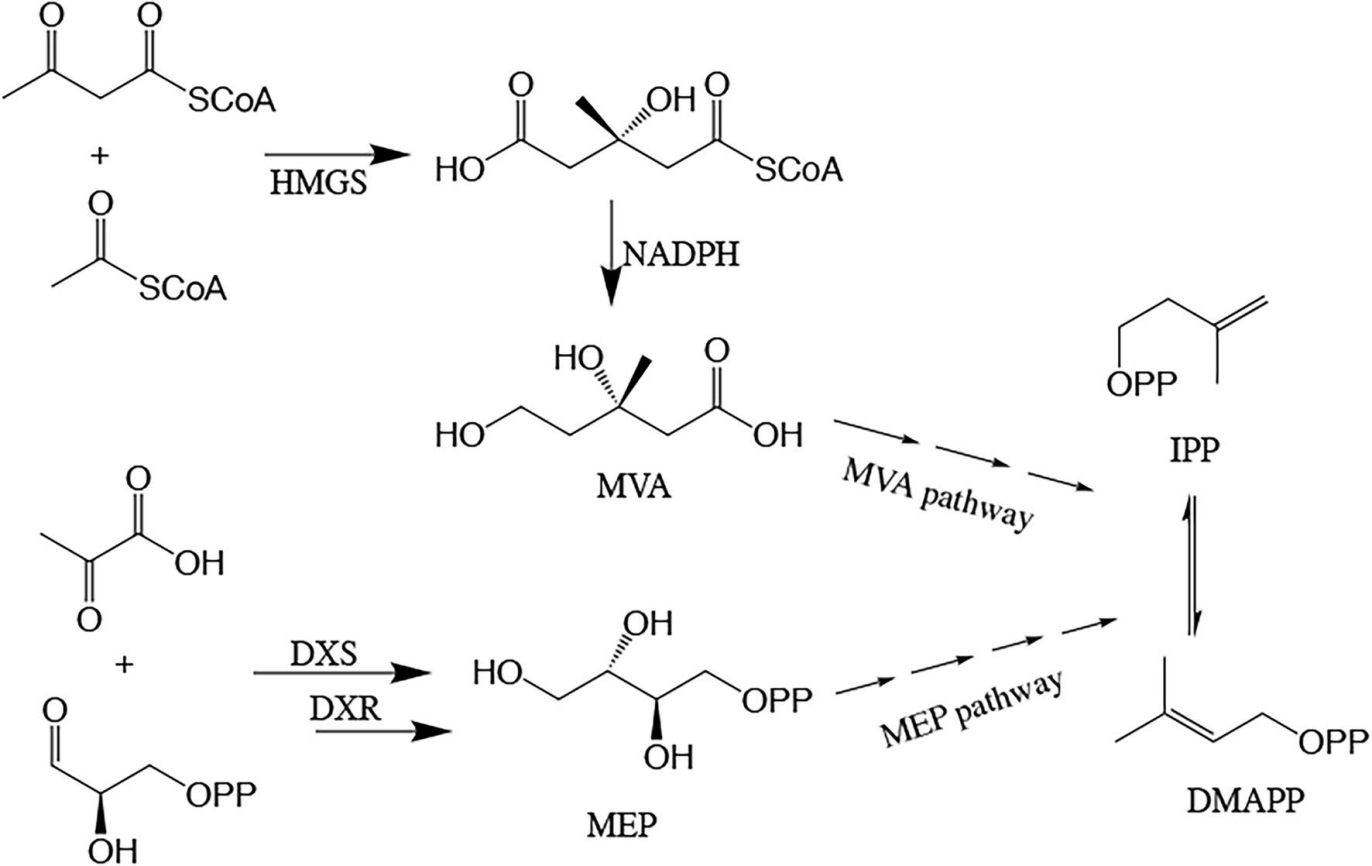
The MVA and MEP pathway of taxol biosynthesis. (Numbers of arrows represent the number of steps. MVA: mevalonate; DOXP: 1-deoxyxylulose 5-phosphate; IPP: isopentenyl pyrophosphate; DMAPP: dimethylallyl diphosphate; HMGS: 3-hydroxy-3-methylglutaryl-coenzyme A synthase; DXR: 1-deoxyxylulose 5-phosphate reductoisomerase; DXS: 1-deoxyxylulose 5-phosphate synthase; NADPH: triphosphopyridine nucleotide; MVA: mevalonate; MEP: 2-C-methyl-d-erythritol-4-phosphate).

The isoprenoids, which are the important precures of taxol biosynthesis, can convert to IPP and DMAPP in higher plants^46,47^. The biosynthesis of IPP and DMAPP includes the MVA and MEP pathways (Fig. 7)^48^. In this study, the genes involved in the MVA pathway were found, such as HMGS, HMGR and HMDR^49^, which catalyze the condensation of acetyl CoA and acetoacetyl CoA to form HMG CoA^49^.

In the plastidial MEP pathway with 1-deoxyxylulose 5-phosphate (DOXP), pyruvate converts into glyceraldehyde-3-phosphate^18,47,48^, and DXR and HDR are the key enzymes^46,50,51^. The expressions of genes coding these two key enzymes were observed through the transcriptome analysis in this study. Moreover, the expressions of these two genes in BT-2 were up-regulated when compared with those of Tax-6.

GPPS, farnesyl pyrophosphate synthetase (FPPS) and GGPPS are the important enzymes for the synthesis of GGPP from GPP and IPP. GGPPS plays an important role in regulating carbon flow^42,52^. In this study, the expressions of the gene coding GPP and GGPPS were found in Tax-6, which showed that the endophytic fungus had a similar isoprenoids synthesis pathway as *Taxus sp.* Although we did not observe the expression of the gene coding FPPS, this also provided the molecular evidence of which the endophytic fungus in *Taxus sp.*, which can produce taxol.

**Figure 8:**
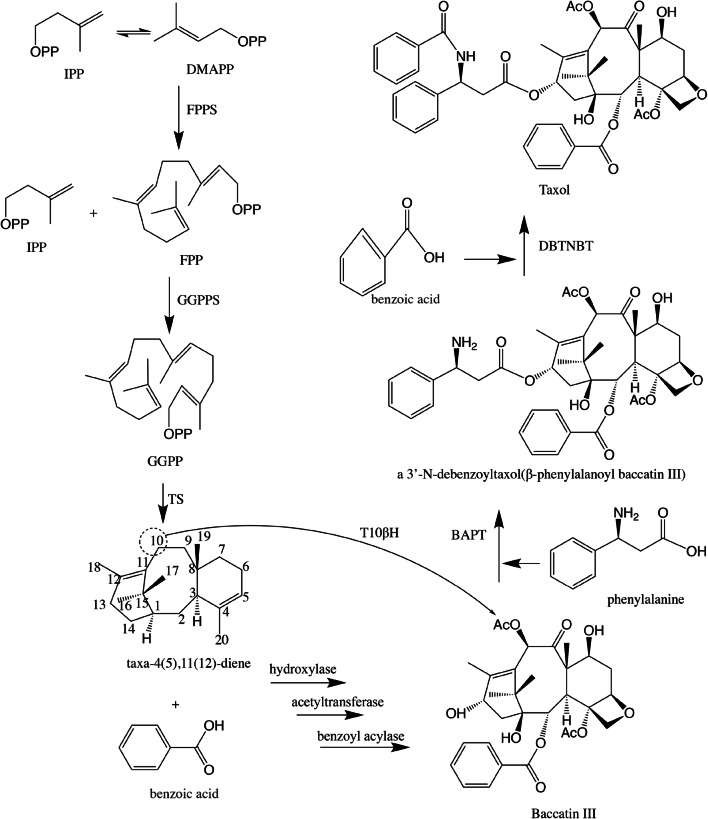
The downstream pathway of taxol biosynthesis. (Numbers of arrows represent the number of steps. DBAT: 10-deacetyl Baccatin III-*O*-acetyltransferase; BAPT: Baccatin III 13-*O*- (3-amino-3-phenylpropanoyl) transferase; DBTNT: 3′-*N*-debenzoyl-2′-deoxytaxol-*N*-benzoyltransferase; IPP: isopentenyl pyrophosphate; DMAPP: dimethylallyl diphosphate; FPPS: farnesyl pyrophosphate synthetase; GGPPS: geranylgeranyl diphosphate synthase; GGPP: geranylgeranyl pyrophosphate).

The cyclization condensation of GGPP is catalyzed to form Taxa-4,11-diene by Taxadiene synthetase (TS) using GGPPS as a raw material^53^. The following synthesis steps of taxol are seven hydroxylation and five acetyltransferase reactions of taxa-4,11-diene, and the benzoyl acylation of side chains on C13 of the taxol backbone (Fig. 8). However, we did not observe an obvious expression of these genes through our transcriptome analysis except for that of T10βH. We suggest that this is the important reason for low taxol production from *A. aculeatinus*.

As a cytochrome P450, T10βH can hydroxylate taxadiene to taxadien-5α-ol and can catalyze taxadien-5α-yl acetate to 5α,10β-taxadien-diol monoacetate. The cytochrome P450-dependent hydroxylation reactions involved in T10βH are the second and fourth specific steps of taxol biosynthesis diverging from primary metabolism (i.e., from GGPP)^54^.

From the transcriptome results, we found the expression of the most upstream genes. However, the genes involved in another key step for taxol biosynthesis, the conversion of GGPP to taxadiene, were not found, nor were the key downstream genes, except for T10βH. When compared with other transcriptome analyses of *Taxus*, our study found relatively fewer enzymes involved in taxol biosynthesis^22,55^. This was likely because transcriptome did not match the genome perfectly, the expression of genes related to taxol production was subjected to environmental changes, and some genes may not be expressed or may only be expressed at low levels until some elicitors are added or are subjected to certain environment stress^56^. We inferred that the absence of those functional genes was possibly because they were not expressed, or because taxol biosynthesis in endophytic fungi is different from that in plants^8^. Additionally, genes involved in the cell cycle were more active in BT-2, which could lead to the production of more biomass in the same culture time, which may also contribute to the production of taxol^57^. The upregulation of genes related to glycine metabolism may also promote taxol production because glycine has been reported to increase taxol production^58^. Finally, we identified NADH dehydrogenase and transcription factors RfeF(AFUA_4G10200)^59^, basic leucine zipper (bZip)^60^, and Specificity protein 1 (SFP1)^61^, which may also contribute to the biosynthesis of taxol, but the mechanism through which this occurs is still unclear.

## Conclusion

We obtained a taxol-producing endophytic fungus, BT-2, by chemical mutagenesis, with a relatively higher taxol yield, conducted transcriptome analysis between two taxol-producing fungi with different taxol production and identified the potential mechanism for the changes in production. The results showed that the expressions of genes involved in the MVA and MEP pathway were almost observed. Moreover, these genes in the mutant strain were up regulated. However, the key downstream genes related to taxol biosynthesis, i.e., TS were hardly found, were far less there were far in fungi than that in *Taxus* plant. In our view, our transcriptome data not only revealed the molecular mechanism by which the endophytic fungus can produce taxol^8^, but also explained the reason why the fungal taxol yields were still low. This study is the first to analyze the transcriptome of taxol-producing fungi and facilitate the promotion of taxol production by *A. aculeatinus*. However, additional studies to clarify the entire pathway of taxol biosynthesis in the endophytic fungi should be performed.

## Supplementary information

Supplementary file1 (DOCX 3749 kb)
