# Supplementary material for: Comparative transcriptome analysis of a taxol-producing endophytic fungus, Aspergillus aculeatinus Tax-6, and its mutant strain
Source: Sci Rep. 2020 Jun 29;10:10558. doi: 10.1038/s41598-020-67614-1 (PMC7324598; doi:10.1038/s41598-020-67614-1)
Supplement: Supplementary file 1 — Supplementary file1 (DOCX 3749 kb) [file 41598_2020_67614_MOESM1_ESM.docx]

**Supplementary Material**

**Comparative transcriptome analysis of a taxol-producing endophytic fungus, *Aspergillus* *aculeatinus* Tax-6, and its mutant strain**

Weichuan Qiao^1*^, Tianhao Tang^1^, Fei Ling^1^

1. Department of Environmental Engineering, College of Biology and the Environment, Nanjing Forestry University, Nanjing，Jiangsu Province, China, 210037

*Corresponding author:

Phone: +86-13851582400;

E-mail: hgqwc@njfu.edu.cn (Weichuan Qiao)

**Table and Figure Captions**

**Table S1.** The qRT-PCR primers of the genes involved in taxol systhesis

**Table S2.** Combined assembly summary of *A. aculeatinus* Tax-6 and BT-2

**Table S3.** Pollution assessment of sequences from *A. aculeatinus* Tax-6 and BT-2

**Table S4.** Proportion of each database annotation

**Table S5.** Genes involved in taxol synthesis in *A. aculeatinus* Tax-6

**Fig. S1.** The death rate of Tax-6 induced by mycostatin.

**Fig. S2.** The coverage of transcriptomic sequencing. (a) *A. aculeatinus* Tax-6; (b) *A. aculeatinus* BT-2

**Fig. S3.** Length distribution of the combined unigenes from *A. aculeatinus* Tax-6 and BT-2

**Fig. S4.** The distribution of GC content of the combined transcripts and unigenes from *A. aculeatinus* Tax-6 and BT-2. (a) transcripts; (b) unigenes

**Fig. S5.** Change in gene expression number in Tax-6 and BT-2

**Fig. S6.** The genes on the terpenoid backbone biosynthesis pathway of BT-2 compared with that of Tax-6 (red: upregulated; green: downregulated; blue: non-differentiated)^1-3^

**Fig. S7.** Genes in the phenylalanine metabolism pathway of BT-2 compared with that of Tax-6 (red: upregulated; green: downregulated; blue: non-differentiated)^1-3^

**Fig. S8.** Genes in the glycine, serine, and threonine metabolism pathway of BT-2 compared with that of Tax-6 (red: upregulated; green: downregulated; blue: non-differentiated)^1-3^

**Table S1.** The qRT-PCR primers of the genes involved in taxol systhesis

| Primer name | Forward Primer | Reverse Primer | Amplification efficiency (%) |
| --- | --- | --- | --- |
| DXR | GCTGCTGACAAGTATCACGG | AGCGACGGTGGAAAATGTTC | 92.6 |
| HMGS | CTACACCG CCCAACAAACACCACCAT | GGCGCTGGAGTAGTAGAACGAGAAGGAG | 94.4 |
| IPPS | GATGATCCACACAATGTCTCTCATTCATGACG | TCGACCGCGCAAGCGGCTGACATGGCAGTAGCC | 93.8 |
| GPPS | GGTTGCCCAAGGTTGGTCTTATTGC | TGAGTAGTAGGCGGTCTTGTACTG | 92.9 |
| GGPPS | TGAATTCCACACGCACAATGGCC | ATAAGCTTCAGTTGGTGCGGGC | 95.6 |
| T10βH | GAGCAACTCCATGGTCTGCT | GAATTTCGTCCAGCTTCGCC | 92.4 |

**Table S2.** Combined assembly summary of *A. aculeatinus* Tax-6 and BT-2

| Assembly | Transcript | Unigene |
| --- | --- | --- |
| All Number | 54793 | 45242 |
| >=500bp | 29812 | 21347 |
| >=1000bp | 19462 | 12425 |
| N50(bp) | 2523 | 2029 |
| N90(bp) | 447 | 354 |
| Average length (bp) | 1239 | 995 |
| All length(bp) | 67895825 | 45016023 |
| Max length(bp) | 15941 | 15941 |
| Min length(bp) | 201 | 201 |

**Table S3.** Pollution assessment of sequences from *A. aculeatinus* Tax-6 and BT-2

| *A. aculeatinus* Tax-6 | | *A. aculeatinus* BT-2 | |
| --- | --- | --- | --- |
| Species | Reads_number | Species | Reads_number |
| *Aspergillus terreus* NIH2624 | 5890 | *Aspergillus niger* | 1287 |
| *Aspergillus aculeatus* | 506 | *Aspergillus japonicus* | 865 |
| *Aspergillus kawachii* IFO 4308 | 175 | *Aspergillus fumigatus* | 170 |
| *Aspergillus clavatus* NRRL 1 | 73 | *Talaromyces flavus* | 20 |
| *Geomyces pannorum* | 21 | *Aspergillus oryzae* RIB40 | 17 |
| *Penicillium chrysogenum Wisconsin* 54-1255 | 21 | *uncultured fungus* | 16 |
| *Scytalidium thermophilum* | 15 | *Paecilomyces variotii* | 13 |
| *Uncultured fungus* | 9 | *Wolfiporia cocos* | 12 |
| *Neurospora crassa* OR74A | 9 | *Penicillium chrysogenum* | 12 |
| *Verticillium ablbo-atrum* VaMs.102 | 4 | *Penicillium ochrosalmoneum* | 8 |
| *Escherichia coli* APEC O78 | 4 | *Ascosphaera torchioi* | 7 |
| *Neurospora crassa* | 4 | *uncultured eukaryote* | 7 |
| *Madurella mycetomatis* | 4 | *Talaromyces stipitatus* ATCC 10500 | 6 |
| *Xylaria* sp.5340 | 3 | *Aspergillus nidulans* FGSC A4 | 5 |
| *Nectria haematococca* mpVI 77-13-4 | 3 | *Penicillium solitum* | 4 |
| *Magnaporthe oryzae* 70-15 | 3 | *Neosartorya fischeri* NRRL 181 | 4 |
| *Prrenophora teres* f.teres 0-1 | 3 | *Aspergillus niger* CBS 513.88 | 3 |
| *Xylaria* sp.5338 | 3 | *Aspergillus candidus* | 2 |
| *Magnaporthe oryzae* | 2 | *Aspergillus bisporus* | 2 |
| *Trichophyton yaoundei* | 2 | *Aspergillus oryzae* 3.042 | 2 |

**Table S4.** Proportion of each database annotation

| Database | Number of Unigenes | Percentage(%) |
| --- | --- | --- |
| Annotated in CDD | 22474 | 49.68 |
| Annotated in KOG | 14410 | 31.85 |
| Annotated in NR | 31167 | 68.89 |
| Annotated in NT | 22794 | 50.38 |
| Annotated in PFAM | 19462 | 43.02 |
| Annotated in Swissprot | 20874 | 46.14 |
| Annotated in TrEMBL | 31026 | 68.58 |
| Annotated in GO | 23462 | 51.86 |
| Annotated in KEGG | 5555 | 12.28 |
| Annotated in at least one database | 34742 | 76.79 |
| Annotated in all database | 3765 | 8.32 |
| Total Unigenes | 45242 | 100 |

**Table S5.** Genes involved in taxol synthesis in *A. aculeatinus* Tax-6

| Gene id | Length | Gene description | Taxonomy |
| --- | --- | --- | --- |
| c12842_g1 | 243 | 1-deoxy-D-xylulose 5-phosphate reductoisomerase (DXR) | *Cladophialophora psammophila* |
| c20284_g1 | 226 | 1-deoxy-D-xylulose 5-phosphate reductoisomerase (DXR) | *Cladophialophora psammophila* |
| c21538_g1 | 626 | 1-deoxy-D-xylulose 5-phosphate reductoisomerase (DXR) | *Cladophialophora psammophila* |
| c35666_g1 | 615 | 1-deoxy-D-xylulose 5-phosphate reductoisomerase (DXR) | *Cladophialophora psammophila* |
| c27345_g1 | 333 | 1-deoxy-D-xylulose-5-phosphate synthase (DXR) | *Beauveria bassiana* |
| c16995_g1 | 416 | 4-hydroxy-3-methylbut-2-en-1-yl diphosphate synthase | *Klebsiella michiganensis* |
| c17343_g1 | 482 | 4-hydroxy-3-methylbut-2-en-1-yl diphosphate synthase | *Klebsiella michiganensis* |
| c22619_g1 | 344 | 4-diphosphocytidyl-2-C-methyl-D-erythritol kinase |  |
| c28734_g1 | 661 | 2-C-methyl-D-erythritol 4-phosphate cytidylyltransferase | *Shigella sonnei* |
| c37814_g1 | 465 | 4-hydroxy-3-methylbut-2-enyl diphosphate reductase | *Shigella flexneri* |
| c39665_g1 | 1301 | 4-hydroxy-3-methylbut-2-enyl diphosphate reductase | *Shigella sonnei* |
| c22619_g1 | 344 | 4-diphosphocytidyl-2-C-methyl-D-erythritol kinase |  |
| c1169_g1 | 2638 | Isopentenyl-diphosphate Delta-isomerase | *Schizosaccharomyces pombe* |
| c12068_g1 | 601 | Isopentenyl-diphosphate Delta-isomerase | *Shigella boydii serotype 4* |
| c29114_g1 | 220 | Isopentenyl-diphosphate delta-isomerase | *Talaromyces stipitatus* |
| c11460_g1 | 279 | Delta(2)-isopentenyl pyrophosphate transferase(IPPS) | *Aspergillus niger* |
| c20682_g1 | 624 | Dimethylallyltransferase | *Shigella dysenteriae serotype 1* |
| c14008_g1 | 560 | Geranyl pyrophosphate transferase （GPPS） |  |
| c12805_g1 | 225 | Geranylgeranyl pyrophosphate synthetase（GGPPS） | *Aspergillus ruber* |
| c42530_g1 | 557 | Geranylgeranyl pyrophosphate synthetase（GGPPS） | *Aspergillus niger* |
| c18500_g1 | 262 | taxane 10-beta-hydroxylase | *Ozonium sp.* BT2 |
| c12174_g1 | 1053 | 3-hydroxy-3-methylglutaryl-CoA synthase, |  |
| c7181_g1 | 1917 | hydroxymethylglutaryl-CoA synthase （HMGS） |  |
| c8758_g1 | 3584 | 1-hydroxy-3-methylglutaryl enzyme A reductase, HMGR) |  |


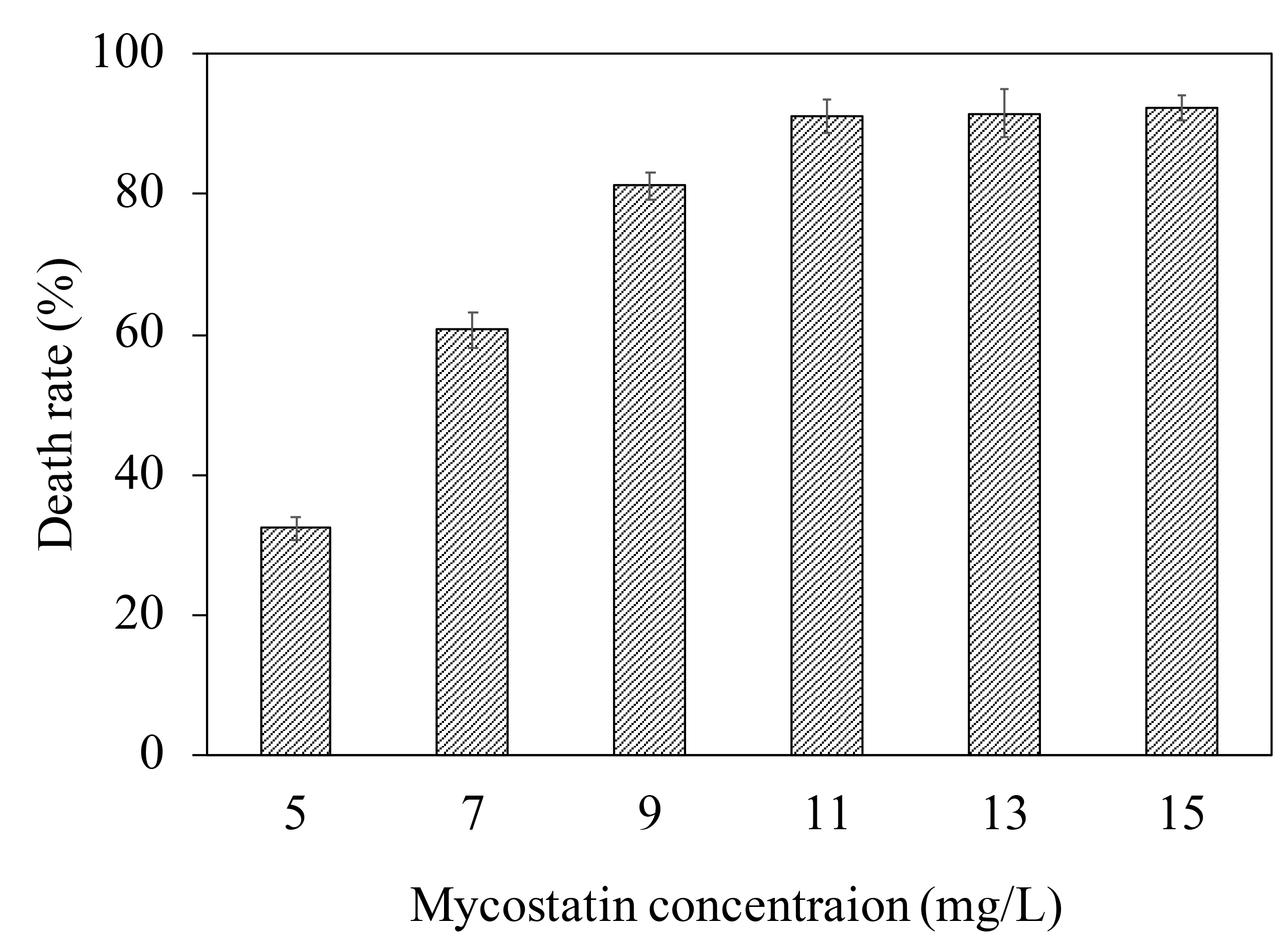


**Fig. S1.** The death rate of Tax-6 induced by mycostatin.


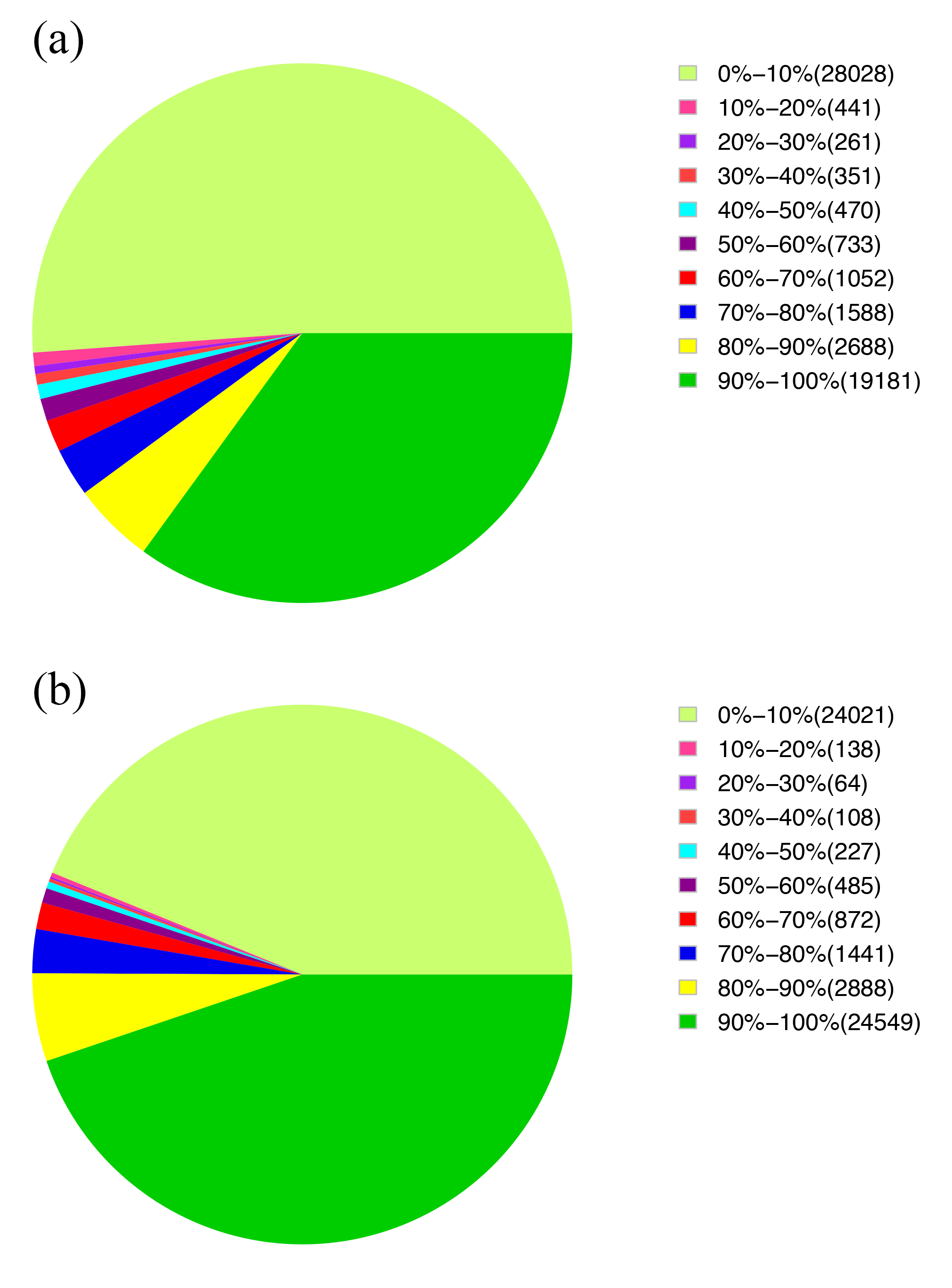


**Fig. S2.** The coverage of transcriptomic sequencing. (a) *A. aculeatinus* Tax-6; (b) *A. aculeatinus* BT-2


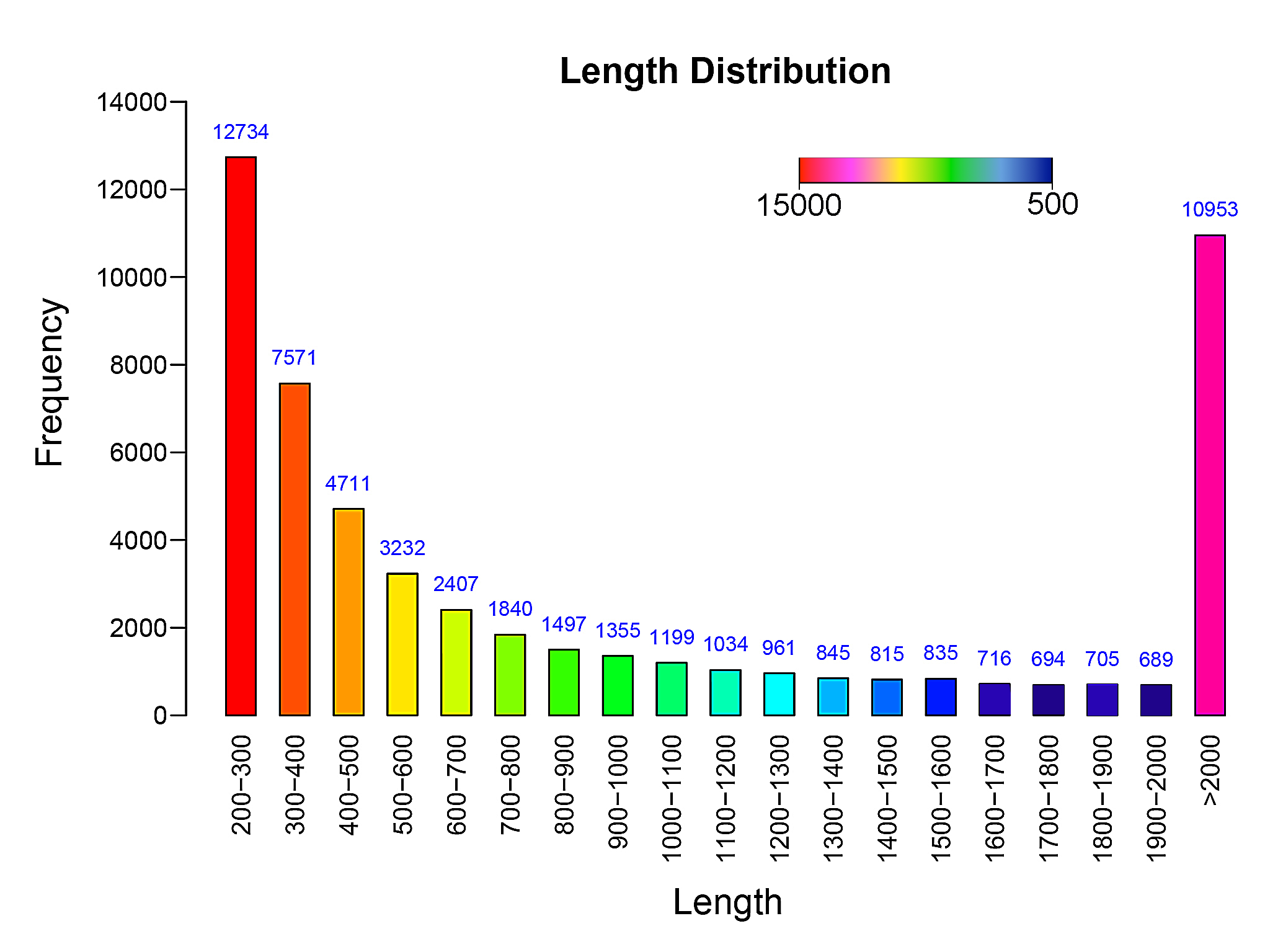


**Fig. S3.** Length distribution of the combined unigenes from *A. aculeatinus* Tax-6 and BT-2

(a)

(b)

**Fig. S4.** The distribution of GC content of the combined transcripts and unigenes from *A. aculeatinus* Tax-6 and BT-2. (a) transcripts; (b) unigenes

**
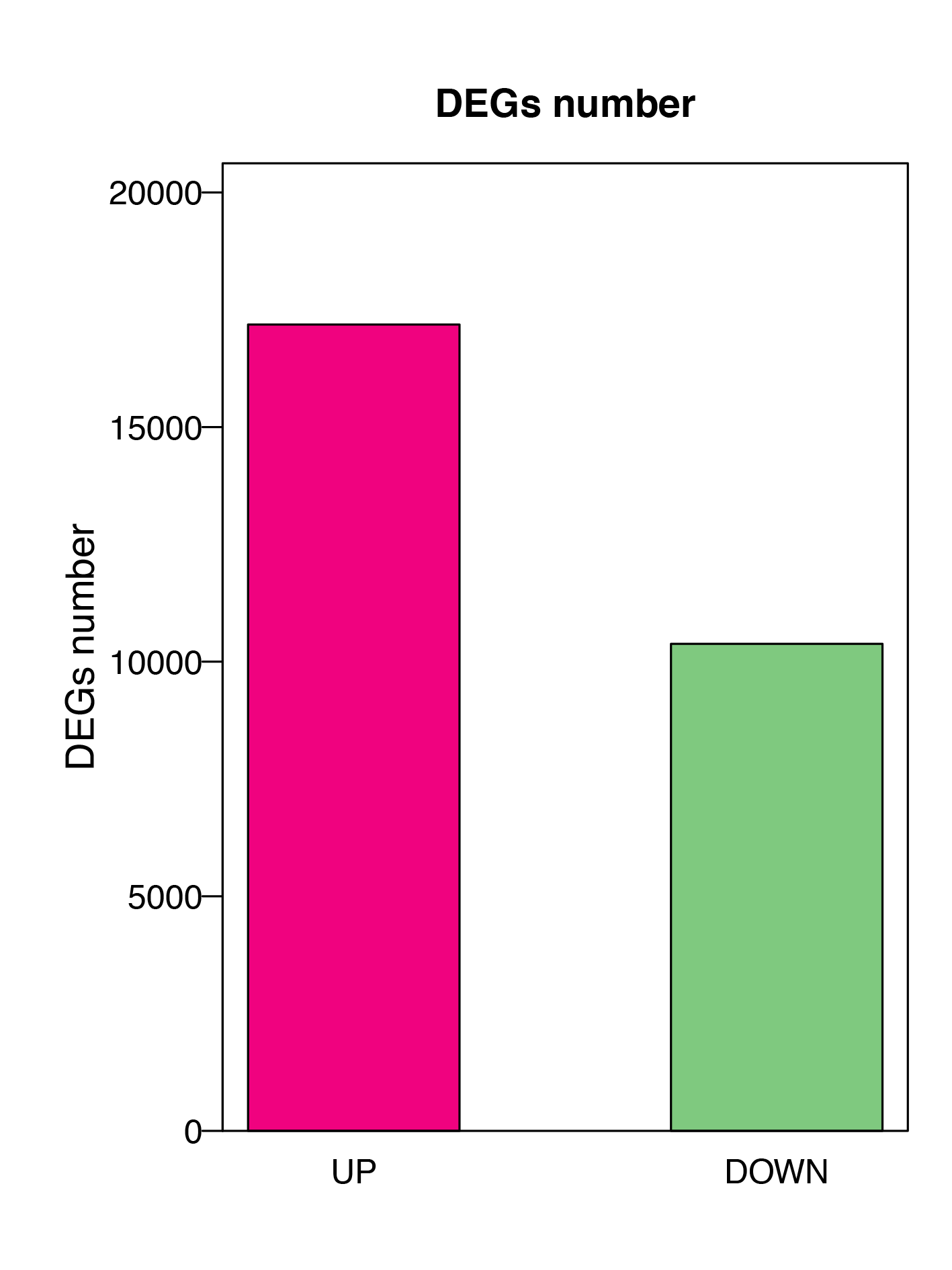
**

**Fig. S5.** Change in gene expression number in Tax-6 and BT-2





**Fig. S6.** The genes on the terpenoid backbone biosynthesis pathway of BT-2 compared with that of Tax-6 (red: upregulated; green: downregulated; blue: non-differentiated)^1-3^





**Fig. S7.** Genes in the phenylalanine metabolism pathway of BT-2 compared with that of Tax-6 (red: upregulated; green: downregulated; blue: non-differentiated)^1-3^





**Fig. S8.** Genes in the glycine, serine, and threonine metabolism pathway of BT-2 compared with that of Tax-6 (red: upregulated; green: downregulated; blue: non-differentiated)^1-3^

**References:**

1 Kanehisa, M., Sato, Y., Furumichi, M., Morishima, K. & Tanabe, M. New approach for understanding genome variations in KEGG. *Nucleic Acids Res.* **47**, D590-D595, doi:10.1093/nar/gky962 (2019).

2 Kanehisa, M. Toward understanding the origin and evolution of cellular organisms. *Protein Sci.*, doi:10.1002/pro.3715 (2019).

3 Kanehisa, M. & Goto, S. KEGG: Kyoto Encyclopedia of Genes and Genomes. *Nucleic Acids Res.* **28**, 27-30, doi:10.1093/nar/28.1.27 (2000).
